# Essay on Growths in the Larynx

**Published:** 1871-10

**Authors:** 


*Essay on Growths in the Larynx.* By Morell Mackenzie, M.D., London, M.R.C.P. Lindsay & Blakeston, Philadelphia, 1871.

This essay contains report and analysis of one hundred cases treated by the author, and a tabular statement of all published cases treated by other practitioners since the invention of the Laryngoscope. Numerous cuts in chromolithography and wood engravings illuminate the book.

The medical profession will appreciate this essay all the more that it is upon a subject which but few practitioners pretend to understand; nearly all are in the position of learners. The Laryngoscope has but recently been introduced into use in the diagnosis of Laryngeal affections, and the illustrations which are here given will serve most admirably to give an idea of what it is expected to show, and what by its aid we may be able to accomplish. Physicians who desire practical knowledge on the subject of Laryngeal outgrowths, then symptoms, appearances, modes of developement, diagnosis and treatment, together with many other allied subjects, will find it open before them in this book most beautifully and plainly.

